# In Situ Collection of Nanoparticles during Femtosecond Laser Machining in Air

**DOI:** 10.3390/nano11092264

**Published:** 2021-08-31

**Authors:** Nithin Joy, Anne-Marie Kietzig

**Affiliations:** Department of Chemical Engineering, McGill University, Montreal, QC H3A 0C5, Canada; nithin.joy@mail.mcgill.ca

**Keywords:** nanoparticles, applied field, applied charge, PLD, laser processing, plume

## Abstract

Nanoparticles generated during laser material processing are often seen as annoying side products, yet they might find useful application upon proper collection. We present a parametric study to identify the dominant factors in nanoparticle removal and collection with the goal of establishing an in situ removal method during femtosecond laser machining. Several target materials of different electrical resistivity, such as Cu, Ti, and Si were laser machined at a relatively high laser fluence. Machining was performed under three different charge conditions, i.e., machining without an externally applied charge (alike atmospheric pulsed laser deposition (PLD)) was compared to machining with a floating potential and with an applied field. Thereby, we investigated the influence of three different charge conditions on the behavior of laser-generated nanoparticles, in particular considering plume deflection, nanoparticle accumulation on a collector plate and their redeposition onto the target. We found that both strategies, machining under a floating potential or under an applied field, were effective for collecting laser-generated nanoparticles. The applied field condition led to the strongest confinement of the nanoparticle plume and tightest resulting nanoparticle collection pattern. Raster-scanning direction was found to influence the nanoparticle collection pattern and ablation depth. However, the laser-processed target surface remained unaffected by the chosen nanoparticle collection strategy. We conclude that machining under a floating potential or an applied field is a promising setup for removing and collecting nanoparticles during the machining process, and thus provides an outlook to circular waste-free laser process design.

## 1. Introduction

Over the past decade, ultrafast laser ablation has emerged as a simple method for the direct generation of nanoparticles. The mechanism of femtosecond laser-assisted material removal is complex and has already been explored in detail [1,2,3]. Many researchers who have studied laser machining in air have commented on the need to clean substrates from nanoparticle debris before further surface analysis or application [4,5,6,7,8]. At higher applied laser fluences, the ejected nanoparticles can even control the laser ablation efficiency due to a strong interaction between the incoming laser beam and the redeposited debris [9]. On the other hand, thus-generated nanoparticles can also enhance the ablation efficiency caused by an enhancement in the local electromagnetic field through the coupling of the incident electromagnetic field with the field induced by the nanoparticles’ surface plasmons [10]. Pulsed laser deposition (PLD) has emerged as a process that makes direct use of the laser-generated nanoparticles in the fabrication of coatings under different processing conditions (vacuum, liquid, and air). Yet, considerable redeposition of the ablated material (nanoparticle debris) on the target makes PLD in air less efficient than when carried out in a vacuum [11,12]. As such, nanoparticles generated by laser machining processes carried out in ambient conditions, which is of special interest for larger-scale industrial applications, are often considered a matter of concern and a useless by-product. Thus, they are either blown off the processing spot by gas flow and/or aspired by a ventilation system, which might or might not be equipped with an appropriate nanoparticle filter. Not surprisingly, such generated nanoparticles pose potential occupational health hazards to researchers and professionals in industry who are exposed to the laser processing environment. Yet, while nanoparticles are a nuisance in some applications, such as in surface micromachining using lasers, they constitute highly desirable materials in others.

The effectiveness of copper as a broad antimicrobial (bacteria, fungi, and viruses) agent has been known since ancient times [13]. Recently, Longano et al. [14] observed the antibacterial activity of laser-generated copper nanoparticles and Sundberg et al. [15] explored the antiviral performance of an aluminum substrate covered in copper nanoparticles. Furthermore, Doremalen et al. [16] and Warnes et al. [17] successfully identified the anti-coronavirus properties of copper and its alloys. In 2020, J. Mostaghimi and his research group showed that copper particles deposited onto both woven and nonwoven fabric, as used for personal protective equipment, such as facial masks, slowed the spread of COVID-19 [18]. Later, in 2021, in an effort to reduce the chance of viral transmission, Jung et al., deposited a thin film of copper (20 nm) on a spunbond polypropylene filter of a KF94 face mask [19]. Similarly, copper, copper-coated substrates, and copper alloys are prime candidates for application on frequently touched surfaces in places with high levels of human circulation, such as hospital utility surfaces and surfaces in public transportation systems in an effort to reduce the spread of infections [20]. Aside from the particular usefulness of copper in antimicrobial applications, owing to their high surface-to-volume ratio and greater surface energy, transition metal nanoparticles in general have gained immense significance as catalysts for a range of organic and inorganic reactions [21]. However, presently, 25 g of copper nanoparticles of, e.g., 25 nm diameter and 99.8% purity is sold for $125, whereas a copper sheet of the same mass can be purchased for less than $2.

Now, considering that nanoparticles are an essential resource for the above-mentioned applications but constitute a poorly managed waste stream in laser surface machining, the paradigm of a circular economy comes to mind [21]. This lets us envision laser micromachining with conscious management of waste/by-product streams from the design phase onwards. The outstanding question is how to best remove and collect nanoparticles, which represent a side product or waste stream during laser processing of, e.g., micro-structured surfaces of interest for various applications in such a manner that the nanoparticles are available as a resource for further applications in, e.g., biotechnology without affecting the machined target surface’s texture or chemistry? Conventional removal techniques that do not fulfil these requirements include ultrasonic cleaning [22], axial flow cyclone cleaning [23], centrifugal spray cleaning, and laser-induced plasma and shock wave removal [24,25].

Zheng et al., introduced a new technique to reduce the amount of Si nanoparticle redeposition onto a femtosecond laser machined target by using an external electric field [26]. In this study, the authors set a minimum voltage of 60 V to 150 V between the two electrodes, and the distance between the electrodes was kept constant at 2 mm. It was found that the particles possessing radii less than 348 nm can be removed from the substrate. The study was limited to a semiconducting Si substrate and a maximal voltage of 150 V.

In this report, we build upon Zheng et al.’s study and investigate nanoparticle collection and removal efficiency during femtosecond laser machining by applying either a high strength electric field or a varying electric field between the collection plate and target. While the collection of Cu nanoparticles is of primary interest, we compare two more target materials of different electrical resistivity. The effect of the polarity of the applied electric charge and the laser scanning direction on nanoparticle collection efficiency is also considered. The comparison and assessments are carried out on the basis of photography of the collection plate upon machining, high-speed videography of the nanoparticle plume deflection, scanning electron microscopy (SEM) and confocal microscopy of the resulting target surface topography.

## 2. Materials and Methods

### 2.1. Materials

Two metals and a semiconductor target were used in this study to generate laser-ablated nanoparticles: copper (99.9% purity, 500 µm thick, McMaster-Carr, Elmhurst, IL, USA), titanium (Grade 2, 99% purity, 500 µm thick, McMaster-Carr, Elmhurst, IL, USA) and silicon (99.9% purity, single step polished, 650 µm thick, University wafers, Boston, MA, USA). The target materials were selected according to their electrical resistivity ranging from low (Cu; ρ = 16.28 nΩ·m) to intermediate (Ti; ρ = 420 nΩ·m) and to high (Si; ρ = 2.3 × 10^3^ Ω·m). Unpolished copper collection plates (99.9% purity, 500 µm thick, McMaster-Carr, Elmhurst, IL, USA) with dimensions of 50 mm × 10 mm were used to collect the generated nanoparticles. All the target samples and the collection metals were ultrasonically cleaned in acetone for 5 min before laser experimentation. The scanning electron microscope images of each of the target material’s surface and the collection plate surface are shown in Figure S1.

### 2.2. Laser Machining

Samples were machined in air using an amplified Ti: Sapphire solid-state laser system (Libra, Coherent Inc., Santa Clara, CL, USA) operating at a central wavelength of 800 nm, a pulse duration of ~100 fs and a repetition rate (*f*) of 1 kHz. The laser energy per pulse (*E*) was reduced to a desired lasing energy using a computer-controlled attenuator composed of a polarizing beam splitter and half-wave plate. The horizontally polarized Gaussian beam (TEM_00_) was then focused down to 40 µm spot diameter ($\omega_{0}$) onto the sample surface using a 200 mm plano-convex lens. The sample was placed at focus on a computer-controlled 3D translation stage (Newport Corporation, Irvine, CL, USA), whose velocity (v) and trajectory were controlled by a trajectory file written in Python (Version 2.7, Python Software Foundation, Wilmington, DE, USA) fed to a stage controller (XPS, Newport Corporation). To enable the comparison across different materials, nanoparticles were produced with a peak laser fluence ($F_{0}=\frac{8E}{\pi \omega_{0}{}^{2}}$) of 4.5 times the ablation thresholds (*F_th_*) of the respective target materials which were raster scanned under the stationary beam. The ablation threshold of each material was experimentally determined by plotting the square of the laser-irradiated line width as a function of the logarithm of the incident laser fluence [27]. The scan line overlap (φ) between two consecutive raster lines, the pulses per spot ($PPS=\frac{\omega_{0}f}{v\;\left(1-\varphi \right)}$), and the machining area were held constant through all experiments at 0.8, 50, and 1 mm^2^, respectively.

### 2.3. Nanoparticle Collection Setup

**Figure 1:**
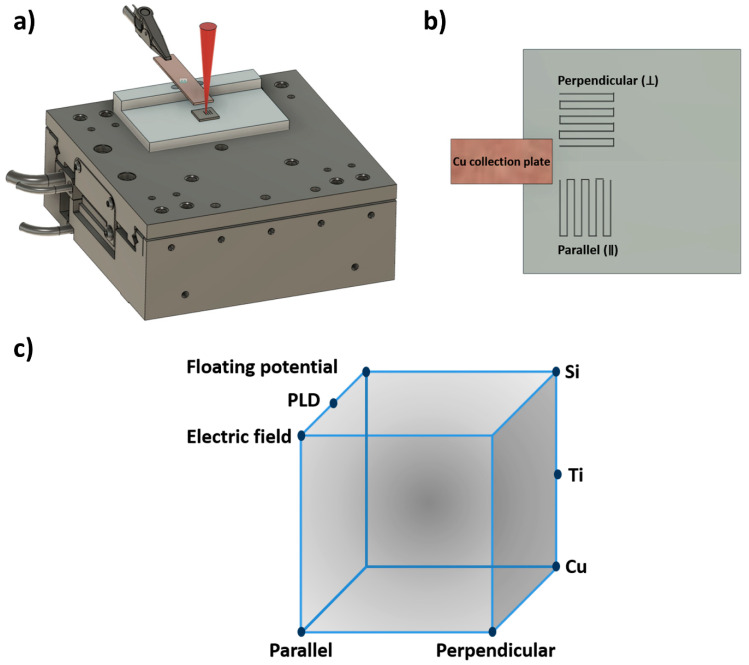
Schematic representation of (**a**) the nanoparticle collection setup, (**b**) the raster laser scanning directions, and (**c**) the experimental space.

The Cu collection plate of 50 mm length and 10 mm width was mounted 5 mm above and parallel to the target sample. The collection plate was connected to a high-voltage DC power supply (Fluke Corporation, Everett, WA, USA), which is schematically represented in Figure 1a. Laser machining experiments were carried out under three different conditions: (1) in ambient air without any charge applied to the collection plate, i.e., corresponding to the typical methodology of pulsed laser deposition (PLD) under ambient conditions; (2) in the presence of a floating potential resulting from an applied potential to the collection plate; and (3) in the presence of an electric field established by applying a potential to the collection plate and grounding the target material. The applied voltage was varied between −2000 V and +2000 V. Furthermore, experiments were carried out in a raster-scan fashion with two different laser scanning directions with respect to the short side of the Cu collection plate (Figure 1b). Figure 1c graphically summarizes the experimental space of our parametric study.

### 2.4. Nanoparticle Plume Videography

The nanoparticle plume was filmed using a high-speed camera (SA5, Photron, Tokyo, Japan) equipped with an 18–108 mm macro zoom lens (Navitar Macro Zoom 7000, Rochester, NY, USA). The camera was temporally synchronized with the femtosecond laser system. The videos were gamma corrected, the region of interest was fixed, and each frame was converted into a .tiff image using PFV software (Version 3.6.9.1, Photron, Tokyo, Japan). These processed images were then fed into a MATLAB code. An intensity threshold was set to distinguish between bright (plume) and dark (background) pixels. Upon identification of the bright pixels and their center points, the plume deflection angle of each frame was determined by fitting the center of bright pixels into a first order polynomial function.

### 2.5. Topographical Characterization

**Figure 2:**
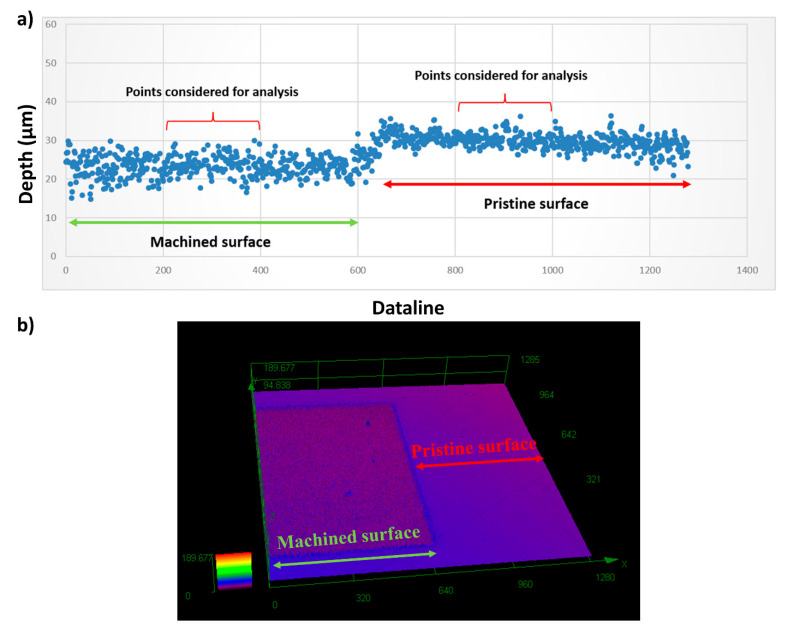
(**a**) Schematic of ablation depth measurement and (**b**) a typical 3D confocal image.

The machined surfaces were imaged with a scanning electron microscope (SEM) (Quanta 450, FEI, Hillsboro, OR, USA) both immediately after laser micromachining and once again after having been ultrasonically cleaned in acetone for 10 min. 3D confocal microscopy (LEXT OLS4000, Olympus Corporation, Tokyo, Japan) was carried out to determine the ablation depth on the target. The depth of all the machined surfaces relative to the pristine surface were measured using the LEXT software (Version 3.1.15, Olympus Corporation, Tokyo, Japan), as shown in Figure 2a. A total of 125 data points were averaged from both the machined surface and the pristine surface at each sample position to estimate the ablation depth. The procedure was repeated for nine different sample positions, the average depth and the standard deviation were obtained. A typical 3D confocal image of a machined surface is depicted in Figure 2b.

## 3. Results and Discussion

All the results presented in the following stem from experiments carried out with parallel laser scanning direction. Section 3.4 will then contrast these findings to those obtained from machining with the perpendicular scanning direction.

### 3.1. Influence of Applied Field/Floating Potential on the Nanoparticle Collection Pattern

**Figure 3:**
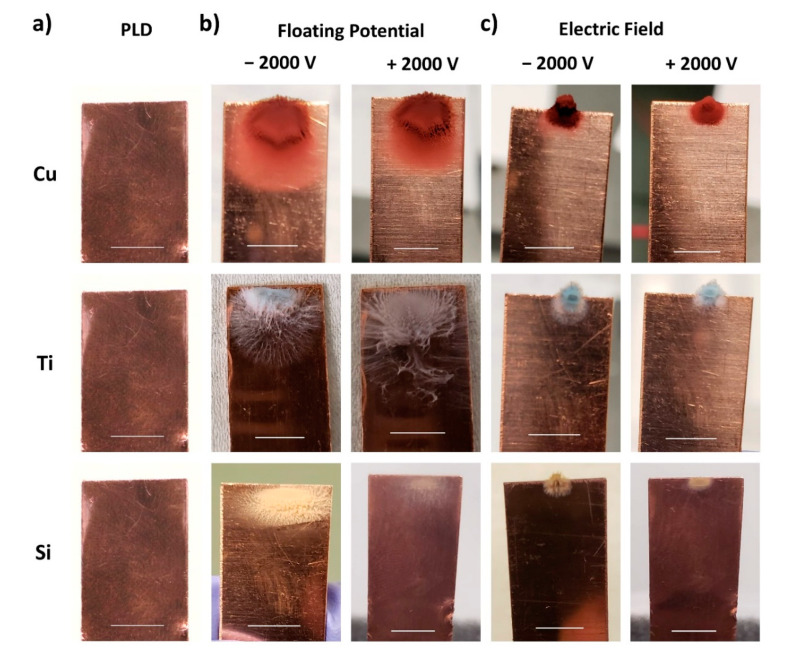
Photographs of the Cu collection plate after the process of laser machining in the parallel laser scanning direction under (**a**) conventional PLD, (**b**) floating potential, and (**c**) external electric field. The scale bars correspond to 5 mm.

Figure 3 shows the photographs of copper collection plates after laser micromachining all investigated target materials under the respective machining conditions. With the conventional PLD setup, no nanoparticles were visible on the collection plate (Figure 3a), which is in contrast to what we observed from the collector plates obtained upon laser micromachining in the presence of a floating potential (Figure 3b) and an applied positive and negative field (Figure 3c). Machining in the presence of a floating potential resulted in a broader nanoparticle collection pattern in contrast to machining in the presence of an applied field, where nanoparticle collection appeared confined to a very small area on the collection plate. This trend was confirmed for both polarities of the applied voltage and all three materials investigated.

From Figure 3b, we also note that the actual pattern of the nanoparticle accumulation on the collector with an externally applied floating potential shows a material dependency. While NPs generated from Cu targets accumulate in a pile on the collector plate, those generated from Ti exhibit a flowery spiderweb structure. A similar observation was reported by Alubaidy et al., but for nanoparticle accumulation on the machined surface [28]. The collection pattern of nanoparticles generated from a Si target also resembles a flower, similar to that observed for Ti, but of different shape and density.

Finally, we note that collection seems to be directly correlated with the materials’ electrical properties (electrical conductivity/resistivity), which are a signature of the number of free electrons present on the material: the seemingly densest nanoparticle packing and maximum collection spread were observed for copper, which has the lowest electrical resistivity among the studied materials, whereas the smallest collection spread with seemingly looser packing was exhibited by Si, which has the highest electrical resistivity. We suggest that the appearance of the nanoparticle packing density and collection spread provide an indication of the absolute number of nanoparticles collected. A study by Irimiciuc et al., showed that an increase in electrical conductivity/decrease in resistivity on metals led to a release of more charge volume and a more intense electrostatic field, which again results in a greater ablation [29]. The highest material removal can therefore be expected for Cu, which fits with our ablation depth results under an applied field (presented in the Supplemental Material), which clearly shows greater ablation depths for Cu than for Ti or Si. Accordingly, lower resistivity leads to more ablation, which results in an increased ejection of particles from the surface, of which a larger number are then also collected on the collector plate.

We acknowledge that photographs merely provide a 2D presentation of a 3D phenomenon, and thus only allow for qualitative comparisons. Ideally, quantitative measures substantiated these conclusions drawn from the “look” of the photographs. Unfortunately, our current experimental setup does not include a sufficiently precise force sensor to make significant in situ high-precision weight measurements of the nanoparticle agglomerates, and any attempts to carefully transfer collection plates from their holder to another instrument to conclusively assess and compare the collected nanoparticle weight proofed ineffective and error-prone. Furthermore, in situ microscopic imaging methods with possible 3D reconstruction would enable a direct analysis of the spatial distribution of the collected nanoparticles.

**Figure 4:**
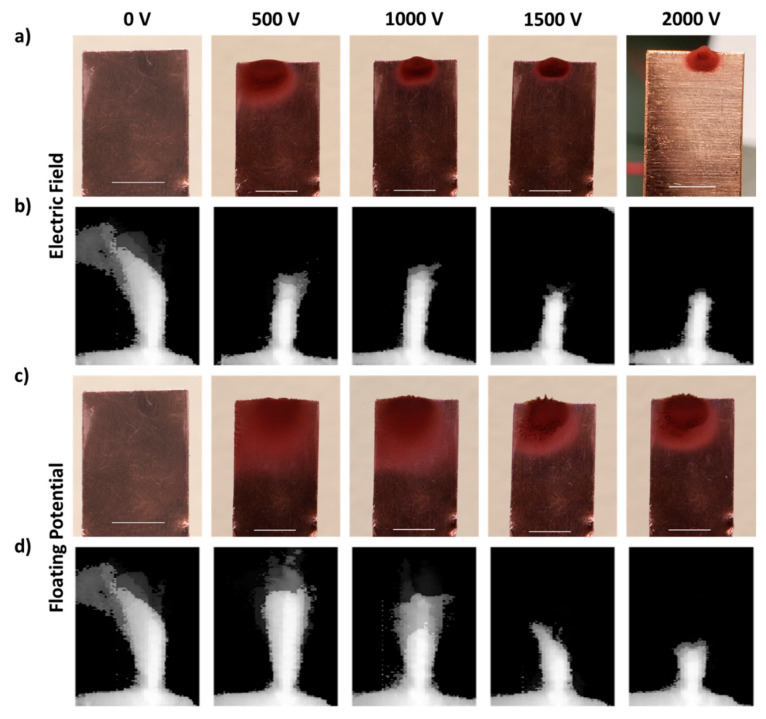
(**a**,**c**) Photographs of the Cu collection plate after the process of laser machining in the parallel laser scanning direction under variable electric field intensities and floating potential intensities respectively; (**b**,**d**) images of nanoparticle plume superimposed of 236 frames extracted from the nanoparticle plume filmed during machining under the respective charge condition in the parallel laser scanning direction. The Cu collection plate was located at the top right of the plume image. The scale bars correspond to 5 mm.

Next, we studied the dependence of nanoparticle collection on the electric field intensity. Figure 4a highlights for Cu exemplarily that the area of the collector plate, on which nanoparticles accumulate, decreases with increasing field intensity. At a higher field intensity, the nanoparticle collection was confined to a very short section of the collector plate’s front edge, which is further discussed in the following section. It is of note that we did not observe a dislodgement of the collected nanoparticles from the collection plate during laser machining.

### 3.2. Nanoparticle Plume Deflection

The nanoparticles plume deflection was visualized using a high-speed camera synchronized with the femtosecond laser system.

**Figure 5:**
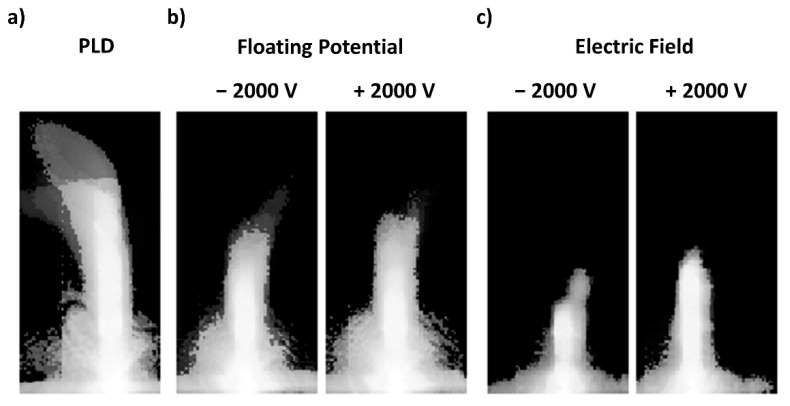
The superimposed images of nanoparticle plume of 236 frames extracted from the nanoparticle plume filmed during the process of laser machining in the parallel laser scanning direction under the (**a**) conventional PLD condition, (**b**) presence of a floating potential, and (**c**) presence of an applied electric field.

Figure 5 demonstrates the behavior of the Cu nanoparticle plume filmed under different machining conditions (conventional PLD, floating potential, and applied field). The laser machining was performed in a raster scan, as shown in Figure 1b, whereas the translation stage moves the target under the stationary laser beam. The stage motion results in a fictitious force on the nanoparticle plume. Thus, for the conventional PLD condition, the high-speed videos show that the nanoparticle plume appears deflected in the direction of stage motion (or opposite of the relative beam motion). Furthermore, plume videography for ablation under PLD conditions illustrates that the nanoparticle plume expands freely in space.

Conversely, a confinement in the nanoparticle plume was observed in the presence of a floating potential (Figure 5b) and applied field (Figure 5c). From a comparison of Figure 5b,c, we note that machining in the vicinity of an applied field results in a more confined nanoparticle plume than what is observed from machining with the floating potential. In the applied field condition, a strong and consistent electric force is applied to the seemingly charged particles (the charge of the nanoparticle thus generated is beyond the scope of the present work, but is under investigation in the context of another study) leading to the visible confinement in the nanoparticle plume, as illustrated in Figure 5c. Similar observations were made for both polarities of the applied voltage (+2000 and −2000 V), yet with slightly more confinement observed for the negative polarity.

When considering the electric field condition, the potential difference across the target and collection plates is definite at ±2000 V. Under such conditions, the electric charge accumulated during the laser ablation process is constant from pulse to pulse, resulting in a steady local electric field which controls the trajectory of the ionized species: the nanoparticles generated during the process of laser ablation. Such a strong and steady local electric field explains the high degree of confinement and the weak angular deflection of the nanoparticle plume (Figure 5c) seen under the electric field condition.

In contrast, neither the conventional PLD condition nor the floating potential conditions are electrically well defined, since the potential difference across the target and collection plates varies from pulse to pulse. The induced electric field will completely depend on the amount of charge left over from the previous pulse (effective field). Thus, this varying local field from pulse to pulse will cause diverse effects on the ionized particle; the behavior of the resulting nanoparticle plume will accordingly be correlated with the varying local electric field intensity. The local electric field for the conventional PLD machining conditions is expected to be weaker than the electric field resulting from the floating potential with an externally applied voltage of ±2000 V because of a relatively small charge accumulation during the laser ablation process. Thus, the differences in local field intensity of an externally applied floating potential compared to that observed under conventional PLD conditions explain the greater nanoparticle plume confinement and the weaker variation in the deflection of the nanoparticle plume. The electric charge accumulated during the laser ablation process is seemingly small; hence, the local electric field induced during the conventional PLD condition is less intense in comparison with the floating potential condition counterpart.

That the applied field intensity influences the nanoparticle plume was further confirmed through plume videography, as seen in Figure 4b. The expansion of the nanoparticle plume decreases with increasing field intensity, where the minimum plume length was observed at our experiment’s maximum applied field intensity of ±2000 V. Therewith, we correlate the confined nanoparticle collection onto a small section of the collector plate edge (Figure 3c and Figure 4a) to the reduced reach of the nanoparticle plume under the application of a higher field intensity. The observations of confinement in the nanoparticle plume and the collection pattern on the collection plate can be explained by the concept of electric field line density. A transverse momentum will be introduced to an ionized species upon collision with a nearby ionized particle, and it will start flying in the transverse direction (the direction perpendicular to the incident laser beam). While in transverse flight, the ionized species will approach the electric field lines between the target and the collection plate and said field lines will deflect the ionized species towards the collection plate. At a higher field intensity, we consider a greater density of the electric field lines; the ionized species will travel a shorter distance in the transverse direction before being deflected towards the collection plate. Conversely, for the lower field intensities, the field lines are less tightly bound, so that the collided particle travel a longer distance in the transverse direction before interacting with the field lines.

A confinement in the nanoparticle collection pattern is also observed under increasing floating potential (Figure 4c). The confinement in the nanoparticle collection pattern on the Cu collection plate observed under various floating potential conditions was seemingly weak in comparison to the respective applied field conditions. At a floating potential corresponding to 500 V, the nanoparticles collected were spread over the entire short edge of the Cu collection plate, while the nanoparticles collected under an applied field corresponding to 500 V were confined to only a small area (refer to Figure 4a). Similarly, an increase in the nanoparticle plume confinement was also observed with an increase in the potential applied to the collection plate similar to the applied field condition (refer to Figure 4d). The differences noticed in the nanoparticle collection patterns on the Cu collection plates at different charge conditions (applied field and floating potential) can thus be explained considering the resulting confinement of the nanoparticle plume. The observations described above are also representative for machining performed in the presence of negatively charged collection plates (Figure S2).

### 3.3. Influence of Applied Field/Floating Potential on Laser-Machined Surface Topography

Having investigated the nanoparticle collection patterns on the collector plates, we now present the effects of the machining conditions on the appearance of the laser-machined target surfaces.

**Figure 6:**
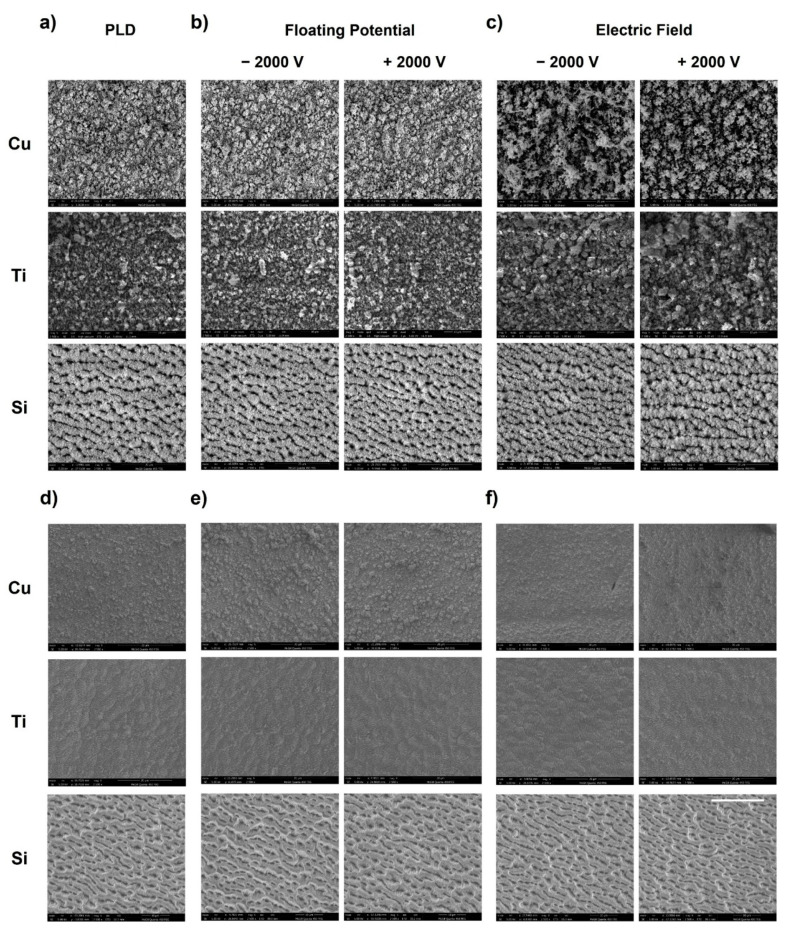
SEM images of Cu, Ti and Si surfaces laser micromachined in the parallel laser scanning direction under conventional PLD conditions (**a**,**d**), in the presence of a floating potential (**b**,**e**), and in the presence of an applied electric field (**c**,**f**), respectively, BEFORE and AFTER ultrasonication. The white scale bar corresponds to 20 µm and applies to all images.

Directly after laser machining, i.e., before ultrasonication, we observe that the actual topography of the machined surface shows some variation with the actual machining conditions (Figure 6a–c). The Cu surface machined in the conventional PLD condition and under a floating potential exhibits nanoparticle agglomerates, while the Cu target resulting from machining in the presence of an external electric field appears covered in less densely packed agglomerates as seen in Figure 6c. The Ti surface machined in the presence of a floating potential and at PLD condition also exhibits a random fibrous structure over the agglomerate structure. Meanwhile, in the presence of an applied field, the surface seems covered with random agglomerate structures. Zimmermann et al., also reported a similar finding [30]. The machined Si surface under all machining conditions shows a comparatively lower nanoparticle cover, as the underlying surface features are still distinguishable (Figure 6). In the presence of an external field, the Si target appears slightly more decorated with nanoparticles compared to the floating potential and conventional PLD condition. The surface topography of the machined surface appears to be similar for both the polarities.

Figure 6d–f show the micrographs of various machined surfaces after ultrasonication. It is evident that the laser machining conditions (conventional PLD setup, applied field or floating potential) for a given material have no significant influence on the resulting surface textures which were previously hidden under agglomerated and non-sintered nanoparticles (refer to Figure 6a–c). This finding is important for the integration of in situ nanoparticle collection in laser surface processing techniques. Furthermore, we realize that while machining with a floating potential or applied field results in the collection of a notable amount of nanoparticles on a collector plate (which is not the case for the ambient PLD condition), this collection is insufficient to remove and collect all nanoparticles produced from the surfaces that were laser-machined with a comparatively high machining fluence of 4.5 times the materials’ threshold fluence. Future work will need to address improving this performance.

While the experimental conditions under investigation here showed no impact of ultrasonication on the laser-induced surface structure after removal of the remaining nanoparticles by, the ablation depth—as determined by confocal microscopy—indicated considerable effects due to the actual experimental conditions. Detailed experimental results are presented in the Supplementary Materials for two target materials: Cu and Ti. The ablation depth differences collected for Si were within the range of error of the measurement technique and are thus not provided here. Interestingly, as shown in Figure S3, the observed ablation depth trends for Cu and Ti are not alike, and no conclusive correlations could be established.

### 3.4. Influence of Parallel and Perpendicular Scanning Directions

**Figure 7:**
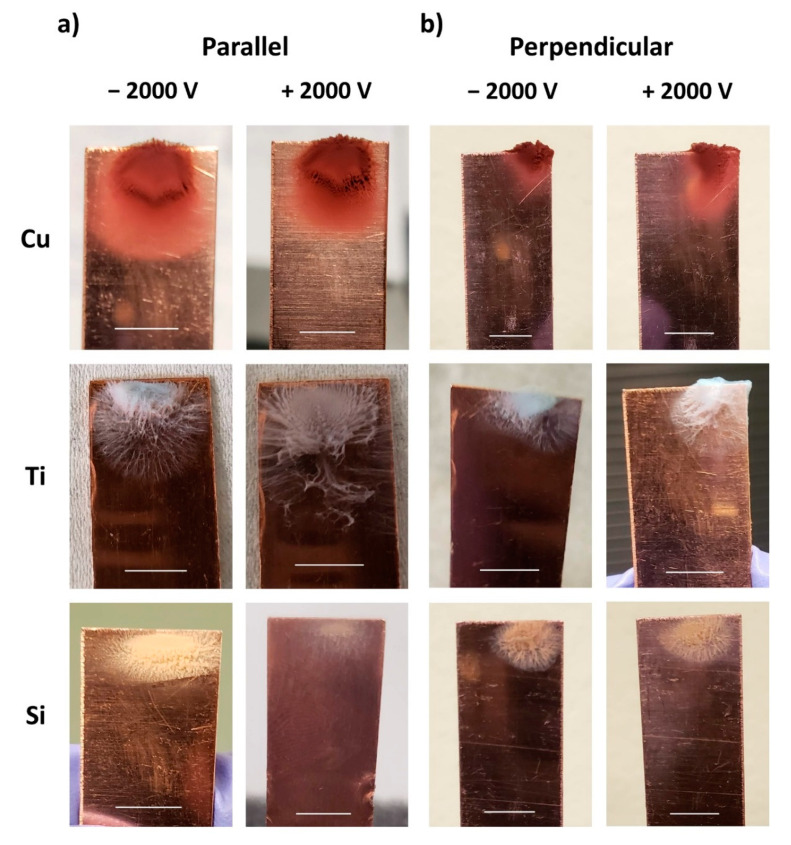
Photographs of the Cu collection plate after the process of laser machining in the (**a**) parallel and (**b**) perpendicular laser scanning direction under the floating potential condition. The scale bars correspond to 5 mm.

The laser machining experiments were performed with two different laser scanning directions (parallel and perpendicular) as depicted in Figure 1b. Up until this point, the discussion has been limited only to the parallel scanning direction. Comparing the photographs of collection plates in parallel (Figure 3c) and perpendicular (Figure S4c) direction, we observe that the nanoparticle collection pattern is comparable and consistent for both laser scanning directions when machined in close proximity to an applied field. For the floating potential, however, machining in the parallel scanning direction results in a greater spread in the nanoparticle collection compared to the perpendicular analog, as shown in Figure 7. For the parallel laser scanning direction, the distance between each long scan line and the short edge of the collection plate is constant (depicted in Figure 1b), which means that the electric force experienced on the laser-generated nanoparticle is also constant throughout each forward (∥−) and backward (∥+) scan. Accordingly, in the very first scan, nanoparticles are attracted and settle somewhat evenly along the short edge of the Cu collector plate. Nanoparticles generated later during the machining of the long scan lines then get attracted and settle onto these first protruding agglomerates, which eventually leads to a broader collection pattern on the collection plate.

Conversely, for the perpendicular case, the distance between the nanoparticle generation sites along the long scan lines and the short edge of the charged collection plate varies. As a consequence, the force acting on the nanoparticles due to the charged collection plate varies throughout the machining of a single long scan line. When the laser incidence position on the target moves away from the short edge of the collector plate, the force decreases; when it approaches the collector plate again in the return scan, the force successively increases. As a result, initially ablated nanoparticles are attracted to a single spot on the collector plate, which is where the initial agglomeration starts and grows into a significant protrusion. The nanoparticles produced during later laterally translated scans will then drive towards this initially generated agglomerate protrusion rather than to the pristine collector plate which is at a slightly farther distance. As a consequence, the perpendicular scanning protocol results in a narrower collection pattern on the Cu collection plate.

Plume videography showed no significant differences between the two scanning protocols (compare Figure 5 with Figure S5).

**Figure 8:**
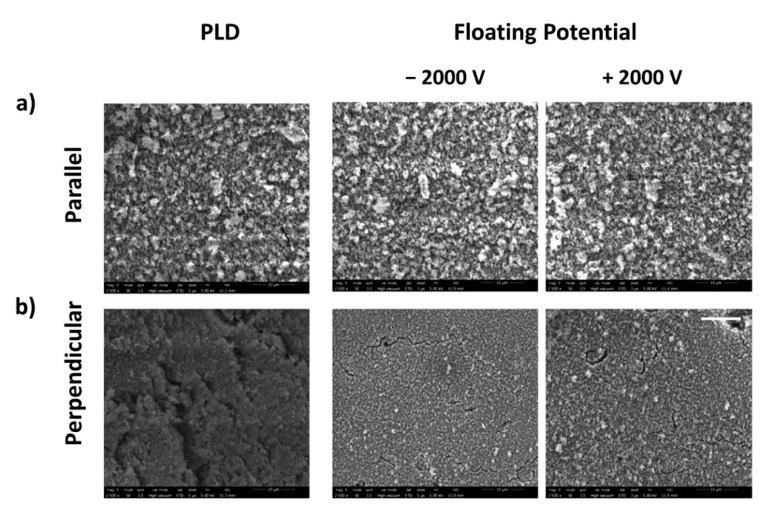
SEM images of Ti surfaces laser micromachined in the (**a**) parallel and (**b**) perpendicular laser scanning directions. The white scale bar corresponds to 10 µm and applies to all images.

The comparison of SEM micrographs of the surfaces machined with parallel and perpendicular scanning direction for a given material after sonication revealed that the scanning direction does not affect the laser-induced structures (compare Figure 6 and Figure S6). Yet, when comparing surface topography directly after laser machining, and thus before ultrasonication, we found differences between the charge conditions and materials. The Cu target topography generally does not differ with laser scanning direction. Similarly, the topography of the machined Ti surface in the presence of an applied electric field is alike for both laser scanning directions. Interestingly, for the Ti target machined in the presence of a floating potential and at the conventional PLD condition, the surface topography differs with laser scanning direction, as depicted in Figure 8. The nanoparticle agglomerates observed on the Ti surface seem more packed when machined in the perpendicular laser scanning direction in contrast to their parallel counterparts. In the case of Si, the surface machined in the parallel scanning direction was decorated with more nanoparticles compared to the surface resulting from perpendicular scanning, irrespective of actual machining conditions. We repeated these experiments multiple times to ensure that these observations were repeatable; at present, we have no explanation for these interesting material-dependent observations.

## 4. Conclusions

In this study, we carried out a parametric study to identify the dominant factors in nanoparticle collection and removal with the goal of establishing therefrom an in situ removal method during femtosecond laser machining. The influence of actual laser machining conditions (conventional PLD condition, floating potential, applied field, laser scanning direction, and the polarity of the applied potential) in removing the laser-produced nanoparticles, collecting them on a Cu collector, and the respective nanoparticle plume deflection behaviors were investigated. The connection between the different surface properties of the machined surface (topography before and after ultrasonication and the ablation depth) and the actual machining conditions were also explored.

Machining in the presence of a floating potential results in a broader nanoparticle collection pattern for all tested targets (Cu, Ti, Si) in contrast to machining in the presence of an applied field, where nanoparticle collection appears confined to a very small area on the collection plate. This observation correlates with a stronger confinement and a weaker angular deflection in the nanoparticle plume that was observed in proximity of an applied field in comparison with the floating potential condition. Under the conventional PLD condition in air, the nanoparticle plume was found to expand freely. The strong and steady local electric field induced under the applied field condition in contrast to the varying local field under the floating potential condition explains the high degree of confinement of the nanoparticle plume and the weak angular deflection of the nanoparticle plume. Furthermore, an increasing confinement of the nanoparticle plume and the resulting nanoparticle collection pattern was also observed with an increase in the applied field intensity and floating potential. Qualitatively judged from the collector plate photographs, the nanoparticle collection quantity directly correlates with the materials’ electrical properties (electrical conductivity/resistivity). Furthermore, the actual accumulation pattern on the Cu collector varies from one material to another, which is particularly evident for the collection patterns obtained upon machining with a floating potential. The topography of the machined surface was examined using SEM and the ablated depth was analyzed with the aid of a confocal microscope. Our results confirm that the surface topography of the machined surface before sonication and the ablation depth strongly depend on the actual charge conditions during machining. Yet, from our experiments, we found no notable impact of the charge condition during laser machining on the resulting laser induced surface textures, which were previously hidden under agglomerated and un-sintered nanoparticles. Whether an electric field or a floating potential is better suited to collect higher amounts of otherwise volatile nanoparticles will be an interesting subject of future studies.

Such experimental efforts might benefit from a setup inside a closed system with a larger collection plate. Ideally, both target and collector are connected to high-precision force sensors. In addition, extremely valuable information would be gained if an alike experimental setup also allows for in situ imaging and sizing of the redeposited and collected nanoparticle clusters.

Based on our findings, we conclude that machining metals and semi-conductors, in particular, under the floating potential or applied field condition with a parallel laser raster scanning protocol is a promising route for removing and collecting nanoparticles during the machining process. The current technique designed for collecting a substantial amount of such laser-generated nanoparticles in air directly during surface machining and patterning without affecting the target surface provides an outlook to circular waste-free process design.

## Data Availability

The data presented in this study are available on request from the corresponding author.

## Supplementary Materials

The following are available online at https://www.mdpi.com/article/10.3390/nano11092264/s1, Figure S1. Scanning electron microscope images of a) the Cu collection plate, and the different target materials before laser machining, i.e., (b) Cu, (c) Ti, and (d) Si. The scale bar indicates 10 μm. Figure S2. (a) & (c) Photographs of the Cu collection plate after the process of laser machining in the parallel laser scanning direction under variable electric field intensities and floating potential respectively (b) & (d) the images of nanoparticle plume superimposed of 236 frames extracted from the nanoparticle plume filmed in the presence of different electric field intensities and floating potential respectively during machining in the parallel laser scanning direction. The Cu collection plate was located at the top right of the plume image. The scale bars are corresponding to 5 mm. Figure S3. Ablation depth of the machined Cu surfaces in the presence of (a) an applied electric field and (b) a floating potential and ablation depth of the machined Ti surfaces in the presence of (c) an applied electric field and (d) a floating potential. The error bars indicate the standard deviation. Figure S4. Photographs of the Cu collection plate after the process of laser machining in the perpendicular laser scanning direction under (a) conventional PLD condition (b) floating potential and (c) external electric field. The scale bars are corresponding to 5 mm. Figure S5. The images of nanoparticle plume superimposed of 236 frames extracted from the nanoparticle plume filmed during the process of laser machining in the perpendicular laser scanning direction under the (a) conventional PLD condition (b) presence of a floating potential and (c) presence of an applied electric field. Figure S6. SEM images of Cu, Ti, and Si surfaces laser micromachined in the perpendicular laser scanning direction in the (a) & (d) conventional PLD condition (b) & (e) presence of a floating potential and (c) & (f) presence of an applied electric field respectively BEFORE and AFTER ultrasonication. The white scale bar corresponds to 20 µm.
