# Supplementary material for: In Situ Collection of Nanoparticles during Femtosecond Laser Machining in Air
Source: Nanomaterials (Basel). 2021 Aug 31;11(9):2264. doi: 10.3390/nano11092264 (PMC8467671; doi:10.3390/nano11092264)
Supplement: Supplementary file 1 [file nanomaterials-11-02264-s001.zip › nanomaterials-1290930-supplementary.pdf]

Supplementary Materials

Article

In Situ Collection of Nanoparticles during Femtosecond Laser Machining in Air

Nithin Joy and Anne-Marie Kietzig *

Department of Chemical Engineering, McGill University, Montreal, QC H3A 0C5, QC, Canada; nithin.joy@mail.mcgill.ca
* Correspondence: anne.kietzig@mcgill.ca

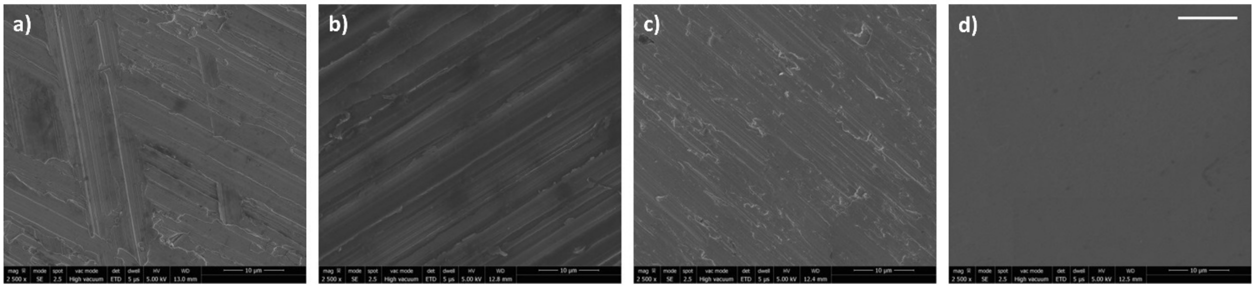

Figure S1. Scanning electron microscope images of a) the Cu collection plate, and the different target materials before laser machining, i.e., b) Cu, c) Ti, and d) Si. The scale bar indicates 10 μm.

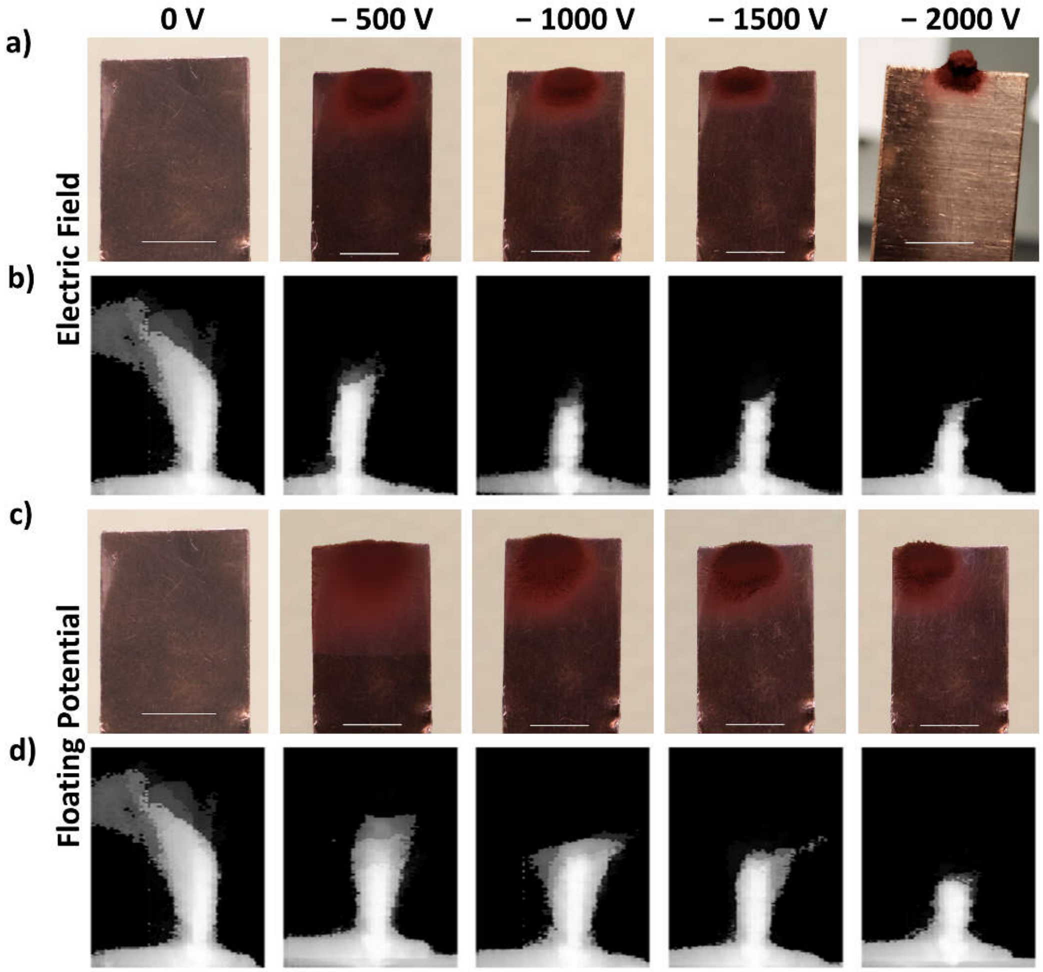

Figure S2. a) & c) Photographs of the Cu collection plate after the process of laser machining in the parallel laser scanning direction under variable electric field intensities and floating potential respectively b) & d) the images of nanoparticle plume superimposed of 236 frames extracted from the nanoparticle plume filmed in the presence of different electric field intensities and floating potential respectively during machining in the parallel laser scanning direction. The Cu collection plate was located at the top right of the plume image. The scale bars are corresponding to 5 mm. .

Supplementary Material 1: Ablation depth analysis

The experimental conditions under investigation here showed no impact on the laser-induced structure after removal of the remaining nanoparticles by ultrasonication. However, the ablation depth - as determined by confocal microscopy - indicates considerable effects of the actual experimental conditions as explained in the following subsections for two target materials: Cu & Ti. Interestingly, the observed ablation depth trends for Cu and Ti are not alike. The ablation depth differences collected for Si were within the range of error of the measurement technique and are thus not provided here. The high reflectivity of the Si surface which results

in a reduction in the absorption of the incoming laser energy contributes to the low ablation depth.

Figure SM1a and b show the ablation depth measured on Cu target surfaces under the different experimental conditions. Upon machining with an external electric field, the largest ablation depth (-14.86 & -15.01 μm respectively for perpendicular & parallel laser scanning direction) was achieved by applying a positive voltage to the collection plate and lowest ablation depth (-9.52 & -6.61 μm respectively for perpendicular & parallel laser scanning direction) was achieved by applying a negative voltage to the collection plate. These results could be explained with the observation of a relatively greater nanoparticle plume confinement at –2000 V in contrast to the positive counterpart (Figure 5b). Whereas, for a floating potential, machining in the presence of a collection plate with a negative potential resulted in the greatest ablation depth (-16.67 & -5.96 μm respectively for perpendicular & parallel laser scanning direction) and machining under the conventional PLD condition resulted in the least ablation (-9.62 & -3.52 μm respectively for perpendicular & parallel laser scanning direction). Alike observations were made for both laser scanning directions. Yet, greater ablation depth was observed for the experiments performed in the perpendicular laser scanning direction in contrast with the parallel laser scanning direction with an exception for Cu under applied field with a positive polarity. Possibly the larger initial protrusion from collection under perpendicular raster scanning promotes stronger removal of the nanoparticles off the machined surface. When considering the case of Cu, surface plasmon resonance might play a role in the observed differences in ablation depth. The collective oscillation of conduction electrons of the Cu nanoparticle clusters redeposited onto the Cu substrate under the incident electromagnetic radiation from the laser has been shown to enhance the incident electromagnetic field and to result in a local amplification in the intensity of the incident laser pulse, which could contribute to an increase in ablation efficiency [1][2]. In addition to the possible SPR assisted enhancement, we observe that the polarity of the externally applied electric field or floating potential significantly affects the ablation, yet we are unable to present a conclusive explanation for the observations.

Figure SM1c and d illustrate the average measured ablation depth of the textured Ti surface machined in the presence of an applied field and a floating potential, respectively. The lowest ablation depth was obtained for the surface that was machined in the conventional PLD condition for both laser scanning directions (-3.45 & -3.85 μm respectively for perpendicular & parallel laser scanning direction). Positive polarity of the applied field resulted in a greater depth (-7.40 & -10.23 μm respectively for perpendicular & parallel laser scanning direction) than negative polarity in both scanning directions (-6.64 & -3.97 μm respectively for perpendicular & parallel laser scanning direction). Similarly, laser machining with a positive floating potential gave a higher ablation depth (-10.76 & -15.66 μm respectively for perpendicular & parallel laser scanning direction) than machining with the negative polarity counterpart (-5.52 & -6.08 μm respectively for perpendicular & parallel laser scanning direction). In general, for Ti a greater ablation depth was observed for laser irradiation performed in the proximity of an applied field/floating potential when compared with the conventional

PLD condition. We attribute this observation to the stronger plume confinement and narrower collection pattern on the collector plate that resulted from machining with the applied field/floating potential when compared with the conventional PLD condition.

Interestingly, an alike clear conclusion cannot be drawn from our experiments with Cu, where the ablation depth under PLD conditions exceeded the same under negative applied field conditions. We presently lack a sound explanation for these observations. Further, we did not observe a visible dislodge of the collected nanoparticles from the collection plate during laser machining. Thus, we conclude for Ti machining that less nanoparticles reached the collection plate under an applied field in contrast to the floating potential. Similar findings, however, for pulsed laser drilling in the presence of an external magnetic field, were reported by Ye et al. and Saxena et al. Here, the application of a magnetic field also resulted in greater confinement of the plasma plume and led to a greater ablation depth [3][4]

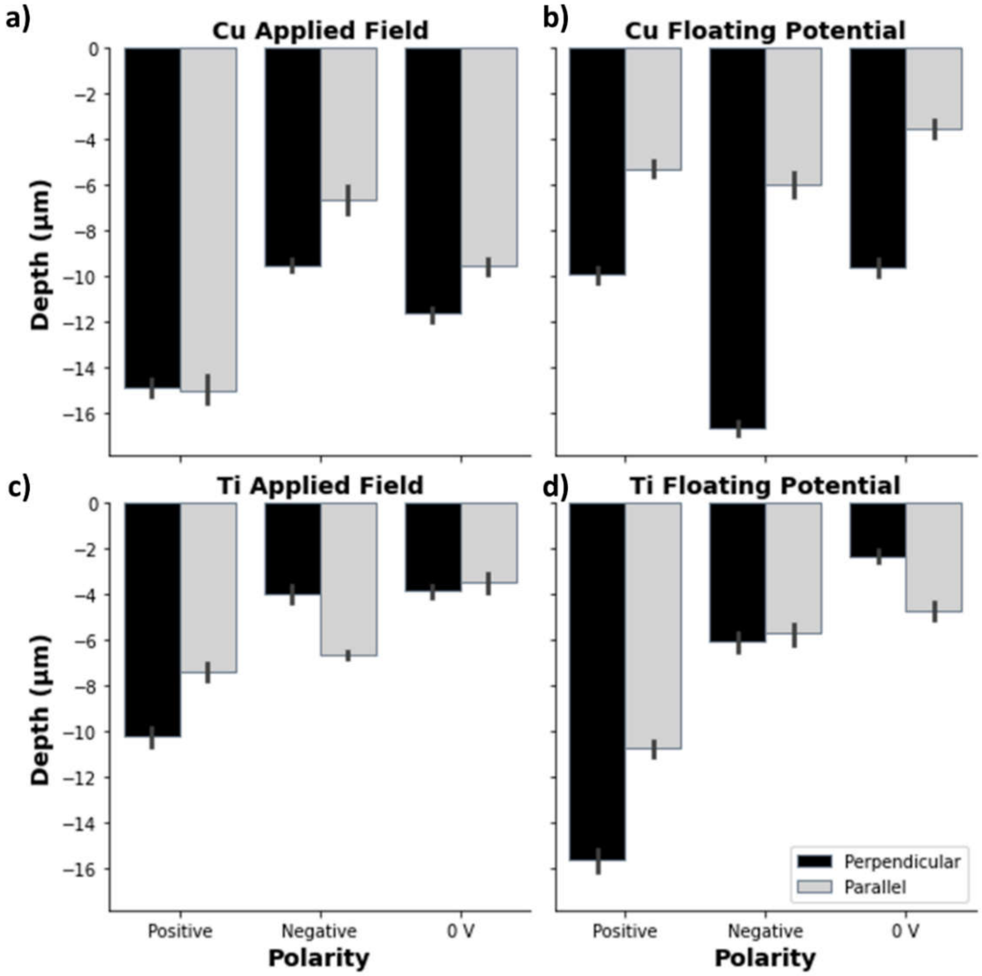

Figure S3. Ablation depth of the machined Cu surfaces in the presence of a) an applied electric field and b) a floating potential and ablation depth of the machined Ti surfaces in the presence of c) an applied electric field and d) a floating potential. The error bars indicate the standard deviation.

Overall, our results for the ablation depth observed on Cu and Ti indicate that standard laser processing with a parallel laser scanning scheme and without applied charges, i.e., a setup similar to conventional PLD carried out in ambient air, results in lowest ablation depth. Further experimentation is required to reach a reliable conclusion on why the laser scanning direction influences ablation depth and whether and how in-situ nanoparticle collection during machining under different charge conditions influences ablation depth.

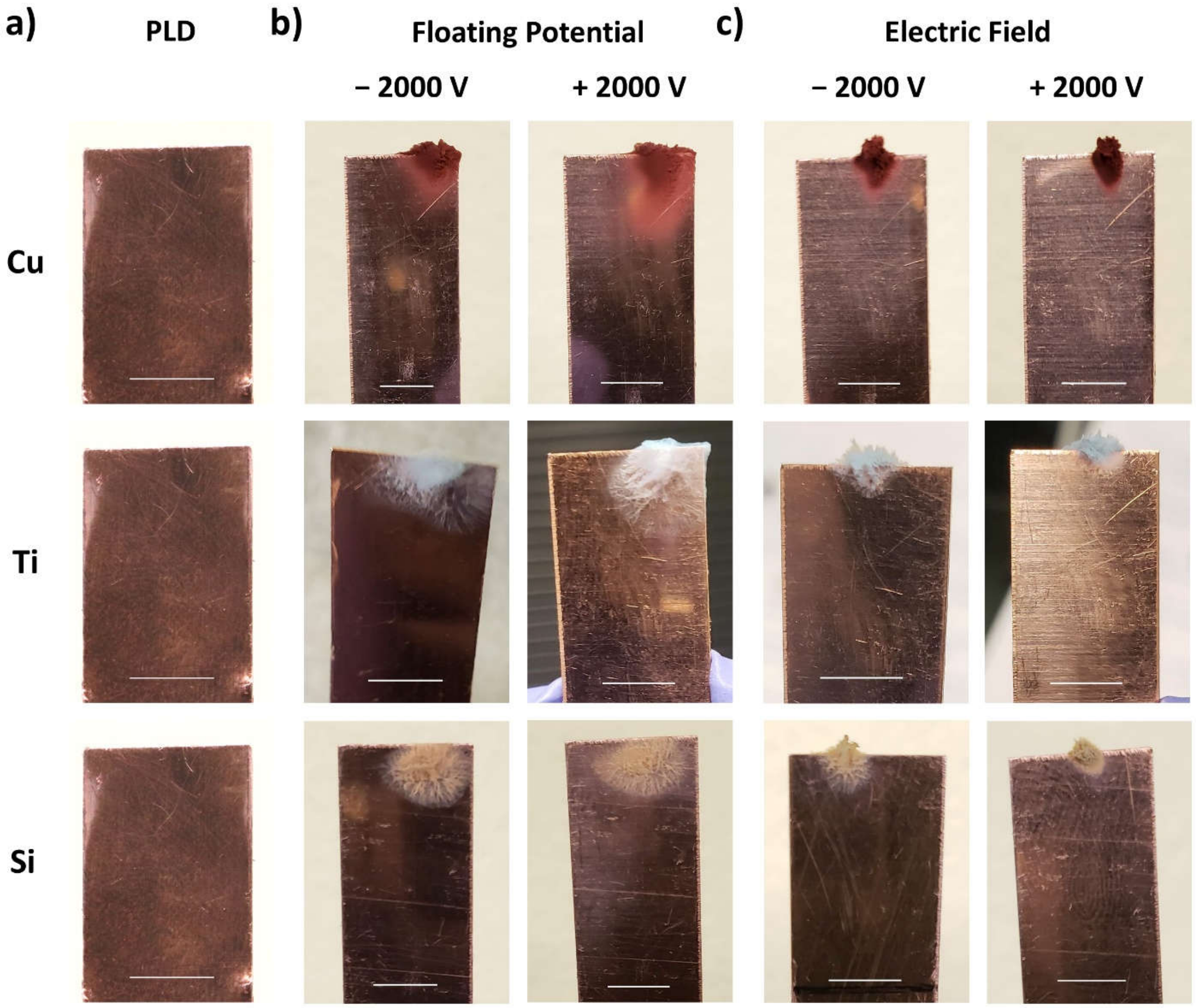

Figure S4. Photographs of the Cu collection plate after the process of laser machining in the perpendicular laser scanning direction under a) conventional PLD condition b) floating potential and c) external electric field. The scale bars are corresponding to 5 mm.

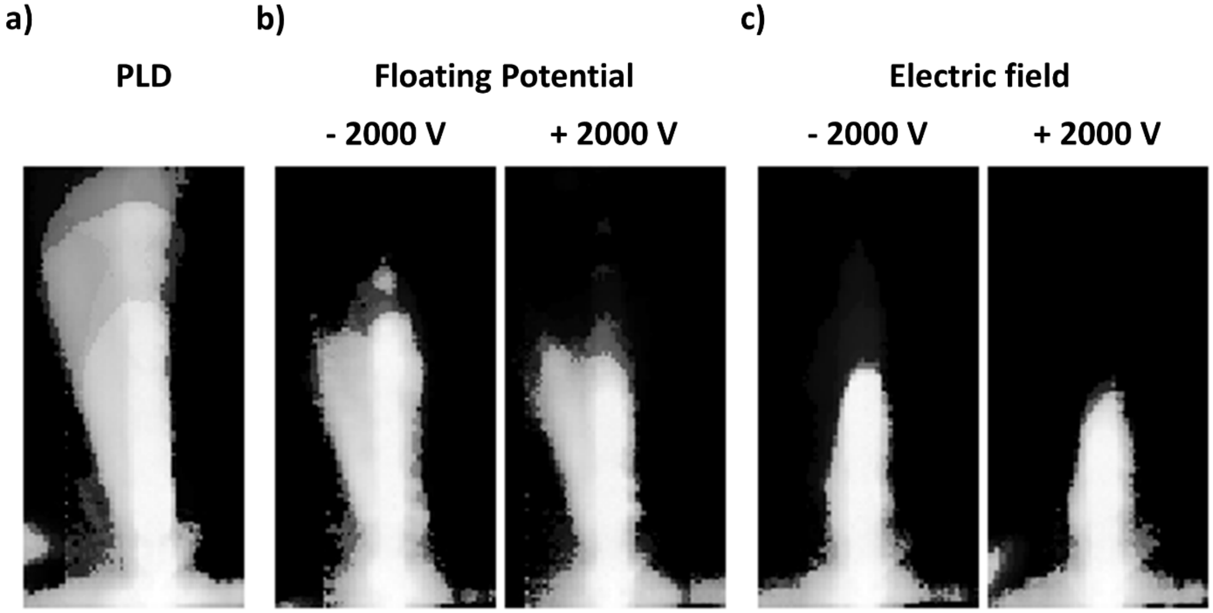

Figure S5. The images of nanoparticle plume superimposed of 236 frames extracted from the nanoparticle plume filmed during the process of laser machining in the perpendicular laser scanning direction under the a) conventional PLD condition b) presence of a floating potential and c) presence of an applied electric field**.**

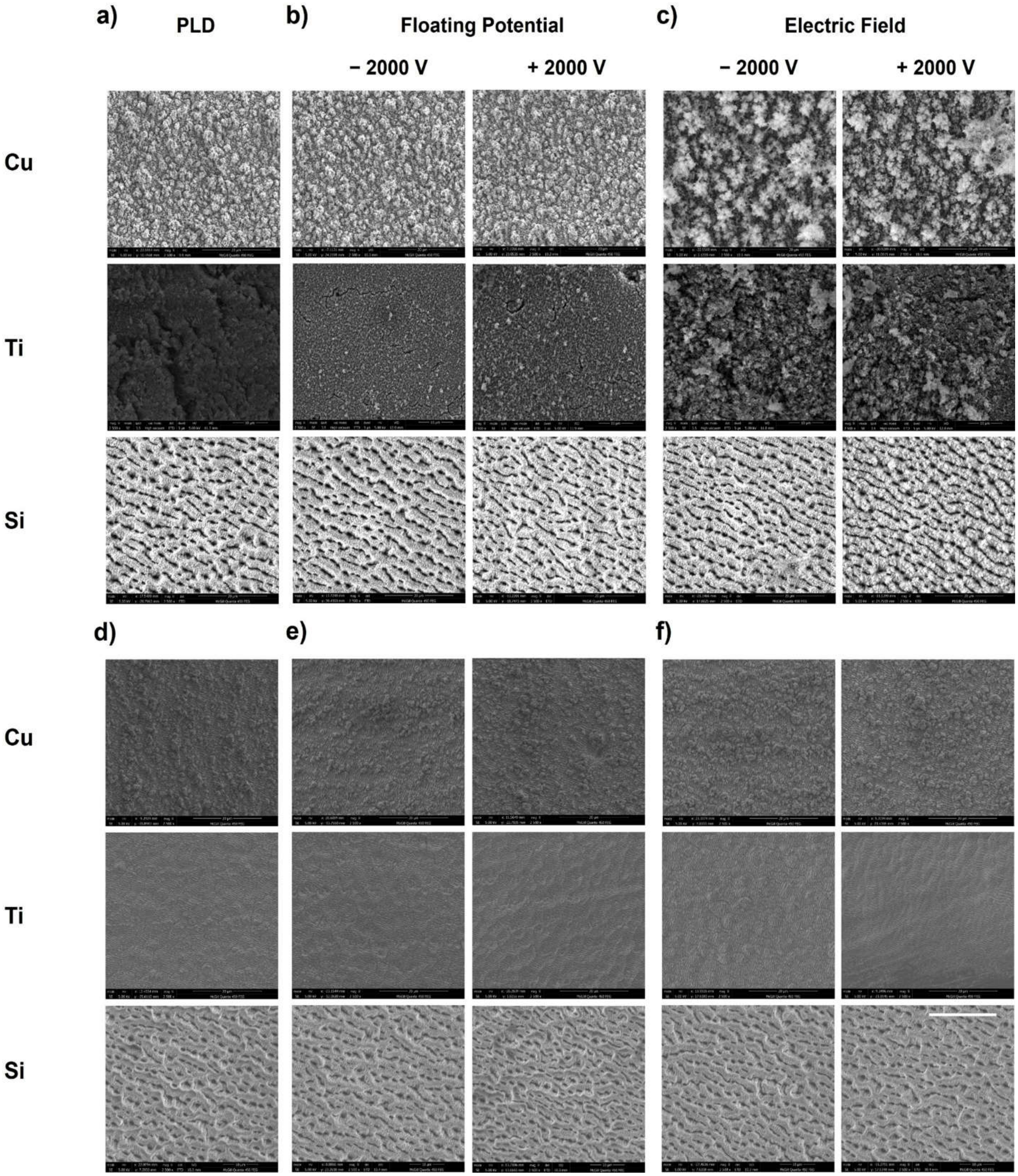

Figure S6. SEM images of Cu, Ti, and Si surfaces laser micromachined in the perpendicular laser scanning direction in the a) & d) conventional PLD condition b) & e) presence of a floating potential and c) & f) presence of an applied electric field respectively BEFORE and AFTER ultrasonication. The white scale bar corresponds to 20 μm.

References

1. De Giacomo, A.; Salajkova, Z.; Dell'aglio, M. A quantum chemistry approach based on the analogy with π-system in polymers for a rapid estimation of the resonance wavelength of nanoparticle systems. *Nanomaterials* **2019**, *9*, doi:10.3390/nano9070929.

2. Dell'Aglio, M.; Alrifai, R.; De Giacomo, A. Nanoparticle Enhanced Laser Induced Breakdown Spectroscopy (NELIBS), a first review. *Spectrochim. Acta - Part B At. Spectrosc.* **2018**, *148*, 105–112, doi:10.1016/j.sab.2018.06.008.

3. Ye, C.; Cheng, G.J.; Tao, S.; Wu, B. Magnetic field effects on laser drilling. *J. Manuf. Sci. Eng. Trans. ASME* **2013**, *135*, 1–5, doi:10.1115/1.4025745.

4. Saxena, I.; Wolff, S.; Cao, J. Unidirectional magnetic field assisted Laser Induced Plasma Micro-Machining. *Manuf. Lett.* **2015**, *3*, 1–4, doi:10.1016/j.mfglet.2014.09.001.
